# Predictors of postoperative pain six months after breast surgery

**DOI:** 10.1038/s41598-023-35426-8

**Published:** 2023-05-23

**Authors:** Delaram J. Ghadimi, Mehdi Azizmohammad Looha, Mohammad Esmaeil Akbari, Atieh Akbari

**Affiliations:** 1 School of Medicine, Shahid Beheshti University of Medical Sciences, Tehran, Iran; 2 Basic and Molecular Epidemiology of Gastrointestinal Disorders Research Center, Research Institute for Gastroenterology and Liver Diseases, Shahid Beheshti University of Medical Sciences, Tehran, Iran; 3 Cancer Research Center, Shahid Beheshti University of Medical Sciences, Tehran, Iran

**Keywords:** Breast cancer, Cancer, Psychology

## Abstract

Breast cancer, with a high prevalence and survival rate, leads to long-term complications. A major sequel is acute or chronic postoperative pain, and we investigated the possible relationship with clinical and psychological variables. Patients undergoing breast surgery filled out the loneliness (ULS-8) and depression (HADS) questionnaires. Patients rated their pain intensity with the Numerical Rating Scale (0–10, NRS) two days, seven days, and six months after surgery. Of 124 patients, the mean age was 45.86 years old, and the pain scores on the second and seventh postoperative days were 5.33 and 3.57, respectively. Sixth-month pain was significantly correlated with the acute scores with a mean of 3.27; and in the multivariate analysis, it was significantly associated with preoperative pain (*p*-value = 0.007), self-reported loneliness (*p*-value = 0.010), and adjuvant radiotherapy (*p*-value = 0.004). In conclusion, loneliness may be a risk factor for postoperative pain in breast surgery.

Breast cancer is the most prevalent cancer, with an incidence of 2.3 million cases per year, increasing with different rates globally. Developments in diagnosis and treatment modalities have led to more survivors living with the consequences of the disease and its treatments^1^.

Breast cancer treatment relies primarily on surgery^2^. Acute and chronic pain are among the most common complications of surgery^3^. Postoperative pain impairs physical, psychological, and social function and reduces the quality of life^4^. Adequate postoperative pain relief reduces recovery time and hospital stay^5^. Also, timely interventions prevent the development of chronic pain, that persists after the usual time for alleviation^6–8^.

We have to better identify the patients at risk for more severe or persistent postoperative pain to manage postoperative pain more effectively^9^. Various characteristics have been investigated as potential risk factors, including loneliness^7^; and is defined as experiencing psychological distress due to a feeling of inadequate social relationships^10^. Loneliness has consequences beyond its psychological burden, such as higher morbidity and mortality^11^. A longitudinal study showed that pain could predict loneliness years later and vice versa, assuming a bidirectional relation between loneliness and pain^12^.

Previous research investigating the relationship between psychological factors and postoperative pain in breast cancer patients had some limitations that needed to be addressed by new studies. First, some studies only assessed pain severity retrospectively^13–15^, which may not capture the full extent of the pain experience. Second, some of the previous studies used small sample sizes^16^, which limits the generalizability of the findings. Third, previous studies have not investigated the potential role of loneliness as a risk factor for postoperative pain in breast cancer patients specifically. To address these limitations, our study tried to use a relatively larger sample size, assess pain levels at multiple time points prospectively, control for potential confounding variables using multiple regression analysis, and specifically investigate the role of loneliness in postoperative pain in breast cancer patients. Therefore, our study aimed to provide new insights into the relationship between psychological factors and postoperative pain in breast cancer patients, and contributes to the existing literature by addressing some of the limitations of previous studies.

## Methods

We included patients undergoing breast surgery at Cancer Research Center, Tehran, between April and October 2020. This study was in accordance with the Helsinki Declaration of 1964, and the Ethics Committee of the Shahid Beheshti University of Medical Sciences approved this study with the code: IR.SBMU.MSP.REC.1398.799. Patients gave their written informed consent to participate in the study.

The exclusion criteria were male gender, illiteracy, prolonged hospital stay after surgery, and loss in the final follow-up. A physician interviewed them within a day before surgery and collected demographic and clinical data by asking the patients or from hospital records. Chronic pain conditions were defined as a history of headache, low back pain, or knee pain (osteoarthritis). In case of uncertain pathological results of biopsies, data was obtained from post-surgery pathology reports.

Depression was evaluated with the depression subscale of the Hospital Anxiety and Depression Scale (HADS), a 4-point Likert scale with seven questions. Patients rated the items based on their emotional state of the last week, and higher scores indicated more severe depression (range 0–21)^17^.

We measured loneliness with a short form of the University of California Los Angeles Loneliness Scale questionnaire with eight items (ULS-8). ULS-8 consists of 6 negative and 2 reverse statements about the general feeling of loneliness and is scored with a Likert scale ranging from 8 to 32. Higher scores indicate a more severe feeling of loneliness^18^. As cancer patients experience different types of loneliness other than social isolation^19^, we added another question to the interview (How often do you feel lonely?) and named it self-reported loneliness, which scored from 1 to 4. The respondent is lonelier as the score increases^20^. The validity and reliability of both questionnaires in Farsi were previously confirmed^21,22^.

We asked patients about their worst pain during the last 24 h. Patients rated their pain in the ipsilateral breast, axilla, chest, and arm by numerical rating scale (NRS), an 11-point measure representing no pain at all with 0 and the worst pain imaginable with 10^23^. We also recorded any pain before the surgery in the areas mentioned above (preoperative pain) and patients’ prediction of the worst pain they would experience right after the surgery (expected postoperative pain). We could not record immediate postoperative analgesic use.

Before discharge, all patients were advised to use Acetaminophen 1000 mg every 6 h or 50 mg Diclofenac every 8 h, or 400 mg Ibuprofen every 8 h if they had intolerable or severe pain. We contacted patients via telephone two and seven days after the operation. On the seventh postoperative day, patients were questioned whether they had used any analgesics or not. We made another phone call during the sixth postoperative month and interviewed patients about their pain and whether they had received adjuvant therapy.

Descriptive statistics were presented using mean ± standard deviation (SD) for numeric variables and frequency (percentage) for categorical variables. The independent t-test and analysis of variance (ANOVA) [The Mann–Whitney test and Kruskal Wallis test in the Supplementary Information] were used to evaluate the relation between pain score and categorical variables by time. The Pearson correlation coefficient was used to examine the association between numeric variables and pain scores. The scatter plot with the linear method was used to show the relationship between numeric variables and pain scores across different times (in the Supplementary Information). The simple and multiple linear regression was used to examine the impact of variables on the pain score across different times. The line plots were used to illustrate the trend of pain scores during time points, and paired t-tests with Bonferroni correction were used to evaluate the difference in pain scores between multiple time points. The mixed-effect model with random intercept and slope was used to assess the longitudinal impact of variables on the pain score. All analyses were performed using R (version 4.1.2) and SPSS (version 26). P-values less than 0.05 were regarded as statistically significant.

## Results

**Figure 1:**
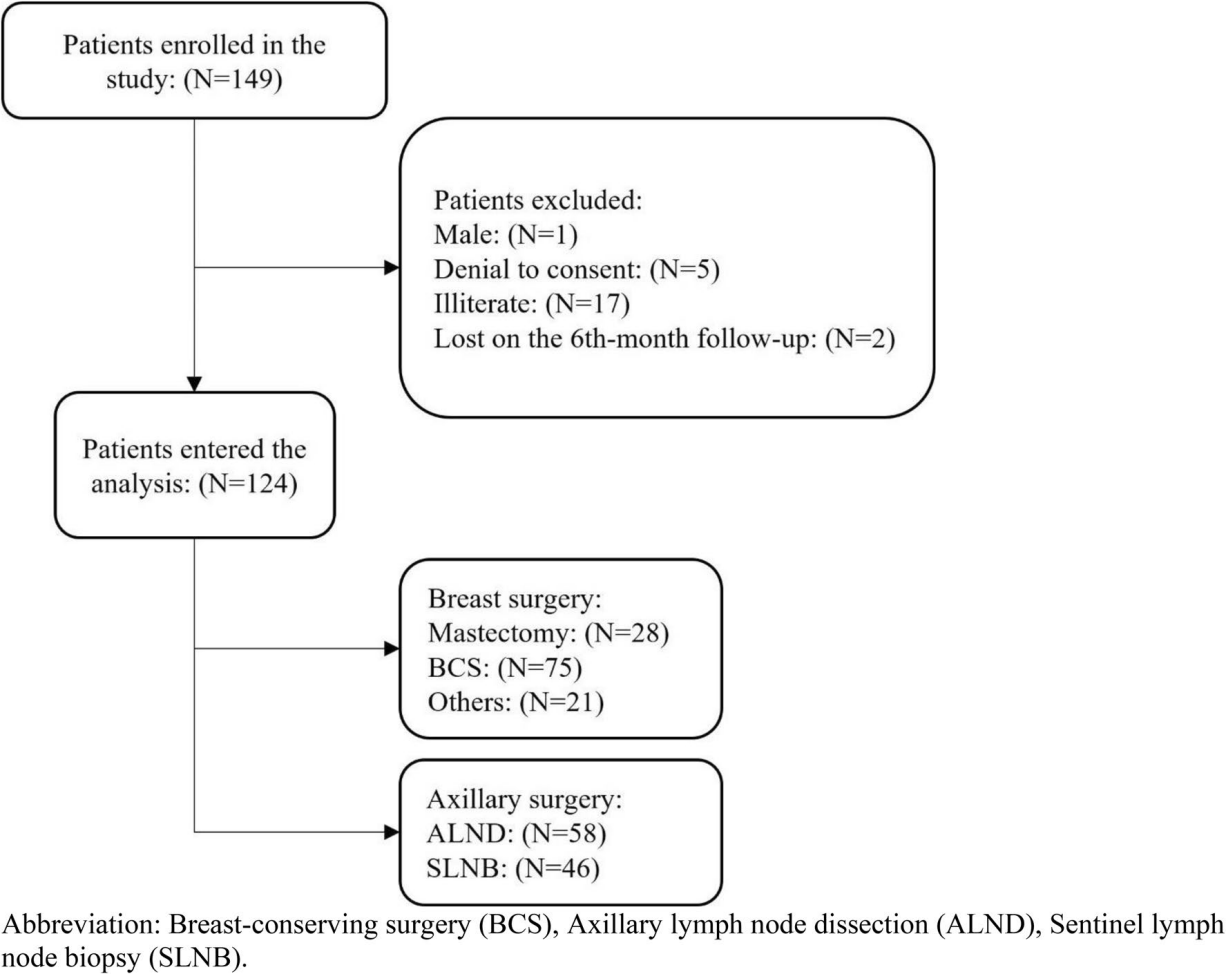
CONSORT diagram of patients enrolled into the study, types of breast and axillary surgery distribution. Abbreviation: Breast-conserving surgery (BCS), Axillary lymph node dissection (ALND), Sentinel lymph node biopsy (SLNB).

**Figure 2:**
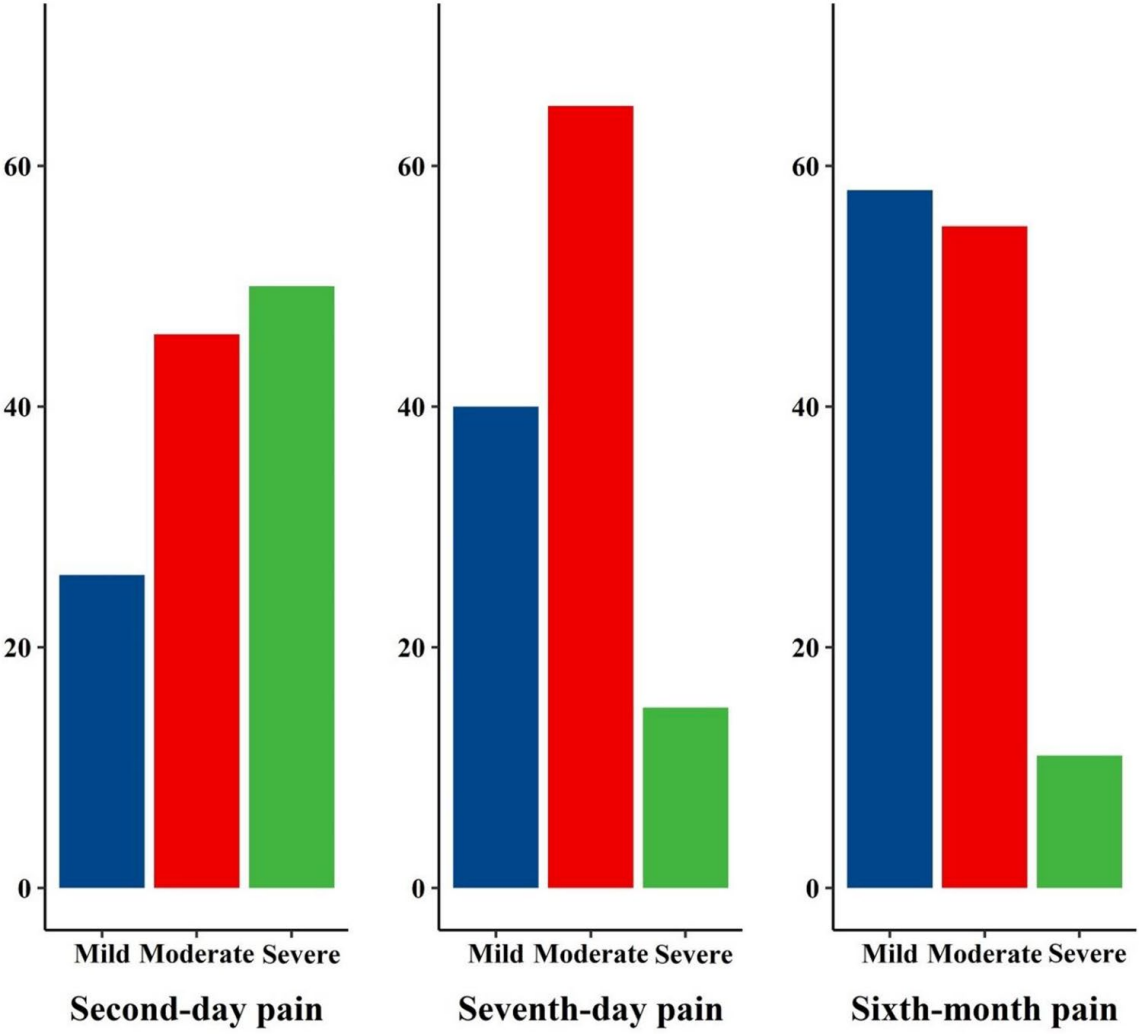
Bar diagram of the frequency of pain levels on the second day, seventh day, and sixth month after surgery (Mild: 0 to 3, moderate: 4 to 6, severe: 7 to 10).

Data on 124 patients enrolled in this study and types of surgery are summarized in Fig. 1. Patients had a mean ± SD age of 45.86 ± 10.55 years (Table 1). Figure 2 shows the distribution of different pain intensities in three endpoints.

**Table 1:** Descriptive statistics of demographic variables and pain scores.

	Frequency (%)/mean ± SD
Age	45.86 ± 10.55
BMI	27.15 ± 4.25
Menopausal age	46.72 ± 4.96
Education	
Under diploma	76 (61.29)
Upper diploma	48 (38.71)
Marital status	
Not-married	16 (12.9)
Married	108 (87.1)
Number of children	
0	16 (12.90)
1	21 (16.90)
2	57 (46.00)
3	23 (18.50)
4	4 (3.20)
5	3 (2.40)
Pain score	
Second day	5.33 ± 2.95
Seventh day	3.57 ± 2.28
Sixth month	3.27 ± 2.51

*BMI* Body mass index.

Different types of breast and axillary surgery led to significantly different pain levels six months after the surgery. The distribution of pain scores in different levels of variables is summarized in Table 2. Patients who used analgesics had significantly more intense pain on the same day and six months later. Also, with every NRS unit increase in second-day pain, the odds of analgesic use significantly increased by 48% on the seventh day; data are shown in the Supplementary information, Table S1.

**Table 2:** The distribution of pain scores in different levels of variables.

Variables	No.	Second-day pain		Seventh-day pain		Sixth-month pain	
		Mean ± SD	*p*-Value	Mean ± SD	*p*-Value	Mean ± SD	*p*-Value
Previous breast operation			0.651		0.758		0.687
No	100	5.27 ± 2.95		3.60 ± 2.23		3.32 ± 2.54	
Yes	24	5.58 ± 3.02		3.44 ± 2.53		3.08 ± 2.46	
Neoadjuvant chemotherapy			**0.003**		**0.006**		0.207
No	88	5.83 ± 2.81		3.89 ± 2.39		3.45 ± 2.66	
Yes	36	4.09 ± 2.97		2.77 ± 1.79		2.81 ± 2.07	
Low back pain			**0.004**		**0.030**		0.054
No	105	5.02 ± 2.89		3.38 ± 2.17		3.09 ± 2.44	
Yes	19	7.14 ± 2.73		4.64 ± 2.65		4.29 ± 2.74	
Knee pain			0.270		0.510		0.223
No	104	5.20 ± 2.89		3.51 ± 2.34		3.15 ± 2.49	
Yes	20	6.00 ± 3.27		3.88 ± 1.95		3.90 ± 2.59	
Headache			0.269		**0.015**		**0.016**
No	97	5.17 ± 3.03		3.30 ± 2.29		2.98 ± 2.37	
Yes	27	5.89 ± 2.64		4.50 ± 2.01		4.30 ± 2.80	
Chronic pain			0.054		**0.013**		**0.041**
No	74	4.91 ± 2.92		3.14 ± 2.20		2.89 ± 2.32	
Yes	50	5.96 ± 2.93		4.18 ± 2.28		3.83 ± 2.70	
Breast surgery*			0.364		0.173		**0.010**
BCS	75	5.38 ± 2.89		3.77 ± 2.25		3.59 ± 2.53^a^	
Mastectomy	28	5.88 ± 3.08		3.80 ± 2.53		3.61 ± 2.45^a^	
Other**	20	4.65 ± 2.88		2.68 ± 1.76		1.75 ± 1.96^b^	
Axillary surgery			0.371		0.145		**0.011**
ALND	58	5.93 ± 2.84		4.00 ± 2.51		3.66 ± 2.34	
SLNB	46	4.78 ± 2.98		3.40 ± 2.06		3.47 ± 2.73	
Pathology			0.228		**0.045**		**0.002**
Benign	19	4.58 ± 2.95		2.61 ± 1.78		1.63 ± 1.94	
Malignant	105	5.47 ± 2.95		3.75 ± 2.33		3.57 ± 2.50	
Analgesic use					** < 0.001**		**0.010**
No	88			2.94 ± 2.06		2.90 ± 2.53	
Yes	32			5.30 ± 1.95		4.23 ± 2.29	
Adjuvant chemotherapy							0.214
No	68					3.01 ± 2.66	
Yes	56					3.58 ± 2.32	
Adjuvant radiotherapy							**0.001**
No	63					2.57 ± 2.54	
Yes	61					3.99 ± 2.29	

The independent t-test was used to evaluate the relation between binary variables and pain across different time points. The ANOVA was used to assess the association between categorical variables and pain scores.

*No.* Number, *BCS* Breast-conserving surgery, *SLNB* Sentinel lymph node biopsy, *ALND* Axillary lymph node dissection.

*Multiple comparisons with the Tukey test determined pain scores at different levels of breast surgery, and different superscript letters show significantly different pain levels.

**Other types of breast surgery rather than mastectomy or BCS.

Significant values are in Bold.

A significant positive correlation was found between the ULS-8 score and pain on the second and sixth postoperative days, along with the sixth-month pain (Table 3). The association between numeric variables and pain scores by time is shown in Fig. S1.

**Table 3:** The association between numeric variables and pain, across different time.

Contrast	Second-day pain		Seventh-day pain		Sixth-month pain	
	r (95% CI)	*p*-Value	r (95% CI)	*p*-Value	r (95% CI)	*p*-Value
Age	−0.10 (−0.27, 0.08)	0.296	−0.07 (−0.24, 0.12)	0.474	**−0.18 (−0.34, 0.00)**	**0.048**
BMI	−0.08 (−0.25, 0.10)	0.402	−0.06 (−0.24, 0.12)	0.530	−0.10 (−0.27, 0.08)	0.283
Menopausal age	0.02 (−0.25, 0.27)	0.912	−0.05 (−0.31, 0.22)	0.732	−0.25 (−0.48, 0.01)	0.061
Preoperative pain	**0.18 (0.01, 0.35)**	**0.044**	0.05 (−0.14, 0.22)	0.620	**0.20 (0.02, 0.36)**	**0.026**
Expected postoperative pain	**0.30 (0.09, 0.49)**	**0.006**	0.18 (−0.04, 0.38)	0.114	0.15 (−0.07, 0.35)	0.168
HADS	**0.18 (0.01, 0.35)**	**0.043**	0.14 (−0.04, 0.31)	0.128	0.09 (−0.09, 0.27)	0.305
ULS-8	**0.23 (0.05, 0.39)**	**0.011**	**0.21 (0.04, 0.38)**	**0.019**	**0.31 (0.14, 0.46)**	**0.001**
Self-reported loneliness	0.09 (−0.09, 0.27)	0.310	0.17 (−0.01, 0.34)	0.066	**0.27 (0.09, 0.42)**	**0.003**
Second-day pain	–	–	–	–	**0.35 (0.18, 0.50)**	** < 0.001**
Seventh-day pain	–	–	–	–	**0.36 (0.19, 0.51)**	** < 0.001**

The Pearson correlation coefficient was used to determine the relation between numeric variables and pain by time.

*BMI* Body mass index, *HADS* Hospital Anxiety and Depression Scale, *ULS-8* University of California Los Angeles Loneliness Scale questionnaire with eight items.

Significant values are in Bold.

In the next step, the impact of variables on pain scores is presented in Table 4. Results showed that a one-unit increase in preoperative pain was associated with a 0.22 increase in second-day pain (*p*-value = 0.044) and a 0.2 increase in the sixth-month pain (*p*-value = 0.026). Increase in ULS-8 was significantly related to higher pain values on the second day (b = 0.13, *p*-value = 0.011), on the seventh day (b = 0.09, *p*-value = 0.019), and on the sixth month (b = 0.014, *p*-value = 0.001). And the self-reported loneliness was associated with higher pain in the sixth month (b = 0.51, *p*-value = 0.003). Other significant effects are shown in Table 4.

**Table 4:** The impact of variables on the pain in different times.

Name	Second-day pain		Seventh-day pain		Sixth-month pain	
	B (95% CI)	*p*-Value	B (95% CI)	*p*-Value	B (95% CI)	*p*-Value
Age	−0.03 (−0.08, 0.02)	0.296	−0.01 (−0.05, 0.03)	0.474	−0.04 (−0.08, 0)	**0.048**
Body Mass Index (BMI)	−0.05 (−0.18, 0.07)	0.402	−0.03 (−0.13, 0.07)	0.530	−0.06 (−0.16, 0.05)	0.283
Menopausal age	0.01 (−0.16, 0.17)	0.912	−0.02 (−0.14, 0.1)	0.732	−0.12 (−0.25, 0.01)	0.061
Preoperative pain	**0.22 (0.01, 0.43)**	**0.044**	0.04 (−0.12, 0.21)	0.620	**0.2 (0.02, 0.38)**	**0.026**
Expected postoperative pain	**0.34 (0.1, 0.58)**	**0.006**	0.15 (−0.04, 0.35)	0.114	0.14 (−0.06, 0.33)	0.168
HADS	**0.21 (0.01, 0.42)**	**0.043**	0.12 (−0.04, 0.28)	0.128	0.09 (−0.08, 0.27)	0.305
ULS-8	**0.13 (0.03, 0.22)**	**0.011**	**0.09 (0.01, 0.16)**	**0.019**	**0.14 (0.06, 0.22)**	**0.001**
Self-reported loneliness	0.2 (−0.19, 0.6)	0.310	0.29 (−0.02, 0.6)	0.066	**0.51 (0.18, 0.83)**	**0.003**
Number of children	0.06 (−0.42, 0.54)	0.819	0.04 (−0.34, 0.41)	0.846	−0.28 (−0.68, 0.13)	0.178
Education; Upper diploma vs. Under diploma	0 (−1.09, 1.09)	0.997	−0.25 (−1.09, 0.59)	0.559	−0.17 (−1.09, 0.75)	0.717
Marital status; Married vs. Non-married	0.42 (−1.15, 1.99)	0.600	0.29 (−0.92, 1.51)	0.634	0.31 (−1.03, 1.65)	0.647
Previous breast operations; Yes vs. No	0.31 (−1.02, 1.65)	0.644	−0.16 (−1.2, 0.87)	0.758	−0.23 (−1.37, 0.9)	0.687
Neoadjuvant chemotherapy; Yes vs. No	**−1.75 (−2.88, −0.62)**	**0.003**	**−1.12 (−2.01, −0.24)**	**0.014**	−0.64 (−1.63, 0.36)	0.207
Low back pain; Yes vs. No	**2.12 (0.67, 3.57)**	**0.004**	**1.26 (0.12, 2.4)**	**0.030**	1.2 (−0.02, 2.43)	0.054
Knee Pain; Yes vs. No	0.8 (−0.63, 2.23)	0.270	0.37 (−0.74, 1.48)	0.510	0.75 (−0.46, 1.96)	0.223
Headache; Yes vs. No	0.72 (−0.56, 1.99)	0.269	**1.2 (0.24, 2.17)**	**0.015**	**1.31 (0.25, 2.37)**	**0.016**
Chronic pain; Yes vs. No	1.05 (−0.02, 2.12)	0.054	**1.04 (0.22, 1.86)**	**0.013**	**0.94 (0.04, 1.84)**	**0.041**
Diabetes mellitus (DM); Yes vs. No	0.34 (−1.24, 1.91)	0.672	−0.58 (−1.8, 0.63)	0.344	−0.38 (−1.72, 0.95)	0.573
Hypertension (HTN); Yes vs. No	−0.03 (−1.75, 1.7)	0.975	−0.95 (−2.32, 0.42)	0.170	0.08 (−1.38, 1.55)	0.909
Pathology; Malignant vs. Benign	0.89 (−0.57, 2.35)	0.228	**1.14 (0.03, 2.26)**	**0.045**	**1.94 (0.74, 3.13)**	**0.002**
Breast surgery						
Mastectomy vs. BCS	0.50 (−0.78, 1.76)	0.446	0.03 (−0.95, 1.01)	0.954	0.01 (−1.04, 1.07)	0.980
Other vs. BCS	−0.73 (−2.18, 0.72)	0.328	−1.10 (−2.21, 0.02)	0.569	**−1.84 (−3.04, −0.64)**	**0.003**
Axillary surgery						
SLNB vs. ALND	−0.57 (−0.15, 0.00)	0.051	−0.30 (−0.77, 0.16)	0.202	−0.10 (−0.59, 0.36)	0.694
Analgesic use; Yes vs. No			**2.36 (1.53, 3.19)**	** < 0.001**	**1.33 (0.32, 2.34)**	**0.010**
Aromatase inhibitor use; Yes vs. No					0.53 (−0.85, 1.9)	0.449
SERM use; Yes vs. No					0.64 (−0.25, 1.54)	0.159
Adjuvant chemotherapy; Yes vs. No*					0.57 (−0.33, 1.46)	0.214
Adjuvant chemotherapy; Yes vs. No**					0.03 (−0.94, 1.00)	0.952
Adjuvant radiotherapy; Yes vs. No					**1.42 (0.56, 2.28)**	**0.001**

The simple linear regression was used to evaluate the impact of variables on the pain in different times.

*HADS* Hospital Anxiety and Depression Scale, *ULS-8* University of California Los Angeles Loneliness Scale questionnaire with eight items, *BCS* Breast conserving surgery, *SLNB* Sentinel lymph node biopsy, *ALND* Axillary lymph node dissection, *SERM* Selective estrogen receptor modulator.

*The subgroup analysis of adjuvant chemotherapy among patients with benign tumor.

**The subgroup analysis of adjuvant chemotherapy for patients with malignant tumor.

Significant values are in Bold.

Table 5 shows the adjusted impact of selected variables on postoperative pain. Preoperative pain (*p*-value = 0.007), self-reported loneliness (*p*-value = 0.01), and adjuvant radiotherapy (p-value = 0.004) had significant effects on sixth-month pain.

**Table 5:** The impact of variables on the pain in different times.

Name	Second-day pain		Seventh-day pain		Sixth-month pain	
	B (95% CI)	*p*-Value	B (95% CI)	*p*-Value	B (95% CI)	*p*-Value
Preoperative pain					**0.31 (0.09, 0.54)**	**0.007**
Expected postoperative pain						
HADS	**0.30 (0.01, 2.58)**	**0.041**	0.20 (−0.03, 2.43)	0.089		
ULS-8						
Self-reported loneliness					**0.53 (0.13, 3.93)**	**0.010**
Chronic pain; Yes vs. No	**1.47 (0.10, 2.84)**	**0.036**	1.02 (−0.14, 2.18)	0.084		
Breast surgery						
Mastectomy vs. BCS	−1.37 (−2.96, 7.23)	0.092			1.11 (−0.12, 2.33)	0.076
Other vs. BCS	0.14 (−5.67, 5.96)	0.961			−0.78 (−5.10, 3.54)	0.721
Axillary surgery						
SLNB vs. ALND	**−1.40 (−2.77, −7.03)**	**0.046**				
Adjuvant radiotherapy; Yes vs. No					**1.59 (0.54, 2.64)**	**0.004**

The multiple linear regression was used to evaluate the impact of variables on the pain in different times.

*HADS* Hospital Anxiety and Depression Scale, *ULS-8* University of California Los Angeles Loneliness Scale questionnaire with eight items, *BCS* Breast conserving surgery, *SLNB* Sentinel lymph node biopsy, *ALND* Axillary lymph node dissection.

Significant values are in Bold.

According to Fig. S2, a significant decline in pain scores was observed between the second day and either the seventh day or sixth month; however, the latter difference was not significant. Table S2 in the Supplementary Information shows the interaction between time and different variables. For instance, the pain declined over time more quickly for the analgesic use group than for those who did not use analgesics.

## Discussion

The present study explored risk factors for postoperative pain in benign and malignant breast neoplasms in a 6-month follow-up. We found that six months after breast surgery, 47.6% of patients still experienced clinically meaningful pain, of which 8.9% had severe pain. In a backward multivariate model, more severe preoperative pain and self-reported loneliness with adjuvant radiotherapy significantly affected the pain intensity. Furthermore, more severe acute pain was correlated with severe chronic pain.

On the second postoperative day, 32 of 122 patients (74.6%) had clinically meaningful pain (> 3 on the NRS scale), and more than half of them suffered from severe pain. This is much higher than the results from previous studies that reported clinically meaningful pain in 45–68% of patients^24–26^. After six months, the mean pain score and number of patients with severe pain decreased significantly. Compared with the first few days, more patients had no or mild pain at the final endpoint. Comparable to an investigation on breast cancer survivors that reported pain resolution in 38% of the patients^27^. In a retrospective study, Fecho et al. found that 8.2% of 196 patients had pain 6 to 12 months following breast surgery, very similar to our findings^13^. The prevalence of chronic postoperative pain in breast surgery ranges from 25 to 60%, based on the study population and variations in defining persistent pain^26,28–30^. In a study of prophylactic mastectomy, 69% of patients reported pain, mainly mild but causing discomfort and annoyance^31^. Our result was not an outlier. We also found a significant correlation between acute and chronic postoperative pain intensity, in line with previous works^26,28,29^.

We noticed a meaningful relation between the sixth-month and preoperative pain in the ipsilateral breast, axilla, chest, and arm. This result ties nicely with other studies^28,32,33^. A survey found no significant association between persistent neuropathic pain after mastectomy and preoperative pain^34^. However, this survey used a narrower definition of chronic postoperative pain and was limited to mastectomy. Also, acute postoperative pain and chronic pain conditions, like headache were significantly correlated, in line with a previous observation^35^. The precise underlying mechanism has not been fully understood yet, but peripheral and central neuroplasticity—meaning changes in the pain threshold in nerves and the brain- is a possible explanation^32,36,37^. It must be pointed out that there is inconsistency within the literature^15,24,26,28,32,38–40^. There is an increased risk of recall bias in retrospective studies, and their results may not be comparable to those from prospective studies. Divergence in pain reporting tools, follow-up periods, definitions of pain (neuropathic nature or just presence of any discomfort or unpleasant feeling), different analytic strategies, and different subsets of patients are some of the possible reasons behind this inconsistency^25,28,30,38,41^.

Of patients, 46% usually felt lonely, similar to other oncology patients^42^. In our study, the feeling of loneliness was significantly associated with acute and chronic pain intensity. Jaremka et al. studied breast cancer patients up to three years after treatment. They investigated immune function -by assessing herpes virus antibody titer- as a possible link between loneliness and health-related outcomes, like pain and depression. They observed a meaningful association between loneliness, pain severity, and depression. They concluded that lonelier patients experience more severe pain while immune dysregulation may be a possible mechanism. Their study evaluated concurrent pain and loneliness, while in longitudinal studies, like ours, loneliness predicted pain for a long time to come^11^. Putting together pain and loneliness are correlated, and the immune system implements this interconnection^10,43,44^.

In our models, depression was associated with pain on the second postoperative day, yet no significant correlation was found between depression and chronic pain. Özalp et al. observed a significant positive relation between the depression score and pain intensity within the first day of mastectomy^45^. The link between depression and acute^26,39^ and chronic pain has been found previously^46,47^, with contrary observations^24,38,48^. In a review article, Ghoneim et al. collected evidence of bidirectional relation between depression and acute or chronic pain. They proposed that pain alters the function of brain cortical regions that play a role in depression; besides, changes in dopamine and serotonin signaling in the brain are present in both conditions and may explain the link between them^49^.

We observed a correlation between younger age and postoperative pain with significance only in the sixth month. Studies have shown the same results and a meaningful relation with acute pain^9,15,25–28,30,39,50^. Possible explanations are senile degeneration in the peripheral and central nervous systems leading to higher pain threshold in the elderly; and more aggressive breast cancer in younger women who need adjuvant treatment more frequently^30,51^.

Soon after the surgery, there was no meaningful difference between the pain intensity in benign or malignant masses. However, on the seventh postoperative day, patients with malignancy experienced more intense pain, and the difference remained after six months, in line with the observations of Kats et al.^25^. We assume that the diagnosis of cancer not only draws attention to the diseased organ but also warrants more aggressive surgeries and adjuvant treatment and causes long-lasting pain.

Patients receiving adjuvant chemotherapy had higher chronic pain intensities, although the difference was not meaningful. Shahbazi et al. did not observe any meaningful relation between the presence of chronic postoperative pain in breast cancer survivors and different chemotherapeutic regimens^34^. Cui et al. found no relation between the history of chemotherapy and chronic pain after breast surgery^15^. In a prospective study of chronic pain after breast surgery, adjuvant chemotherapy and chronic pain were not associated^52^. There were also observations of increased chronic pain incidence with adjuvant chemotherapy^26,53^. Because of these inconsistencies, we did not enter chemotherapy in our final model.

Many patients with breast cancer receive radiotherapy, and most experience complications like pain, whether because of skin reactions or damage to vessels and nerves^54,55^. Adjuvant radiotherapy was associated with more intense chronic pain in all our models. As Fig. S2 shows, in patients who did not receive radiotherapy, chronic pain was significantly lower than acute pain scores, and the recipients of radiotherapy had an increase in their pain scores. We found studies with consistent results^14,28,53,56,57^. Although, there were opposing conclusions as well^15,30,50^.

Our study had several limitations; a small number of patients and a short follow-up period among them. We could not record anesthetic drugs and analgesics used during surgery and the first 24 h after surgery. Also, we did not document radiotherapy doses and chemotherapy regimens. We assessed depression and loneliness once during the cohort and did not consider the possible changes in those feelings during the course of treatment. Furthermore, we did not differentiate between neuropathic and non-neuropathic pain, which increased the number of patients labeled with clinically meaningful pain in the sixth month. Unfortunately, we could not include patients who received intraoperative radiotherapy and compare it with conventional adjuvant radiotherapy.

Another potential limitation of the current study is the limited number of investigated psychological variables. In order to keep the length of the questionnaire to a minimum and maintain accurate engagement^59^, only depression and loneliness were included as self-reported psychological factors, and we did not evaluate other potential psychological determinants of pain, like anxiety^45^. While these variables are important and have been linked to various health issues, the exclusion of other psychological factors may have limited the scope and generalizability of the findings. Future research may benefit from including a broader range of psychological variables in the questionnaire to provide a more comprehensive understanding of the relationship between psychological factors and health outcomes. Moreover, in spite of the widely accepted and validated nature of the worst pain-NRS as a measure of pain intensity in clinical research, we acknowledge that our study's reliance on this single measure as the primary outcome may have limited our ability to capture the full scope of pain-related outcomes. In addition, we recognize that by not including other measures such as average pain-NRS, pain-related symptoms, disabilities, and quality of life, we may have missed important information related to patients’ pain experience. However, we designed our study protocol and sample size calculation based on the primary outcome measure, and incorporating additional measures would have required a larger sample size and increased the study complexity. Therefore, while we acknowledge these limitations, we believe that our study still provides valuable insights into pain outcomes in our specific population. Future studies should consider including a more comprehensive set of measures to better capture the complexity of pain-related outcomes.

Another limitation of the study is the lack of in-person follow-ups, which may have affected the accuracy and completeness of data collection on postoperative complications. Although the researchers attempted to collect data through phone interviews, this method may not have been as reliable or comprehensive as in-person examination. Consequently, the study did not include complications^58^ such as infection, seroma formation, and lymphedema or the disease stage in the model, which may have affected the clarity of the definitions of postoperative complications. To address these limitations, future studies could consider alternative methods for collecting data on complications, such as telemedicine or video consultations, to ensure the completeness and accuracy of the data. Our article is among the few to investigate the impact of loneliness on postoperative pain in breast surgery. We also included benign pathologies since they are common indications of breast surgery. We also evaluated pain scores prospectively to avoid recall bias^28^.

## Conclusion

We identified several determinants of acute and chronic postoperative pain. Although in the multivariate analysis, different explanatory variables were found for acute and chronic postoperative pain. First, the more severe the pain is in the first few days after the surgery, the more probable the patient will experience clinically meaningful pain six months later. Second, psychological factors are critical: depression is associated with increased acute pain, and loneliness results in a higher prevalence of pain six months after surgery. Finally, analgesic use in the first postoperative days may be an early predictor of chronic pain. All indicate a broad range of factors that affect postoperative pain. Our findings suggest that clinicians should give more attention to patients' psychological distress to minimize physical complications, and further studies with larger cohorts and wider scopes should be designed.

## Supplementary Information

Supplementary Information.

## Data Availability

This article is taken from the disease registry titled “Breast cancer clinical registry in Iran”, supported by the deputy of research and technology at Shahid Beheshti University of Medical Sciences (http://dregistry.sbmu.ac.ir). Data generated or analyzed during the study will be available from the corresponding author on reasonable request.
