# Supplementary material for: Predictors of postoperative pain six months after breast surgery
Source: Sci Rep. 2023 May 23;13:8302. doi: 10.1038/s41598-023-35426-8 (PMC10205704; doi:10.1038/s41598-023-35426-8)
Supplement: Supplementary file 1 — Supplementary Information. [file 41598_2023_35426_MOESM1_ESM.pdf]

Predictors of postoperative pain six months after breast surgery

Supplementary Information

Table S1. The relation between pain on the second day and analgesic use on the seventh day

Variable	OR (95% CI)	P-value
Pain on the second day	1.48 (1.23, 1.79)	<0.001

Note: Dependent variable is analgesic use

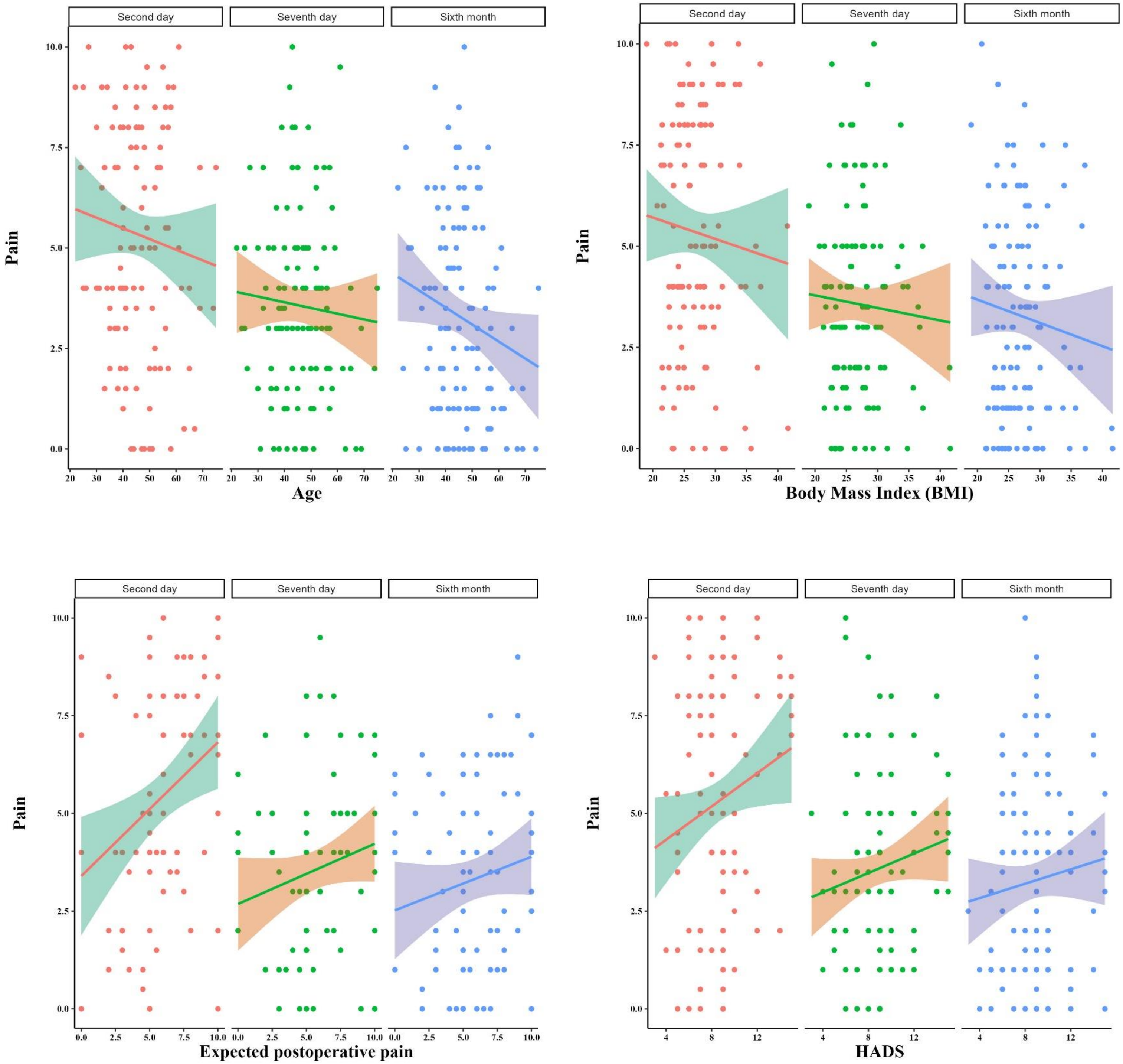

Figure S1. The relation between pain at different times (second day, seventh day, and sixth month) and variables using the linear method

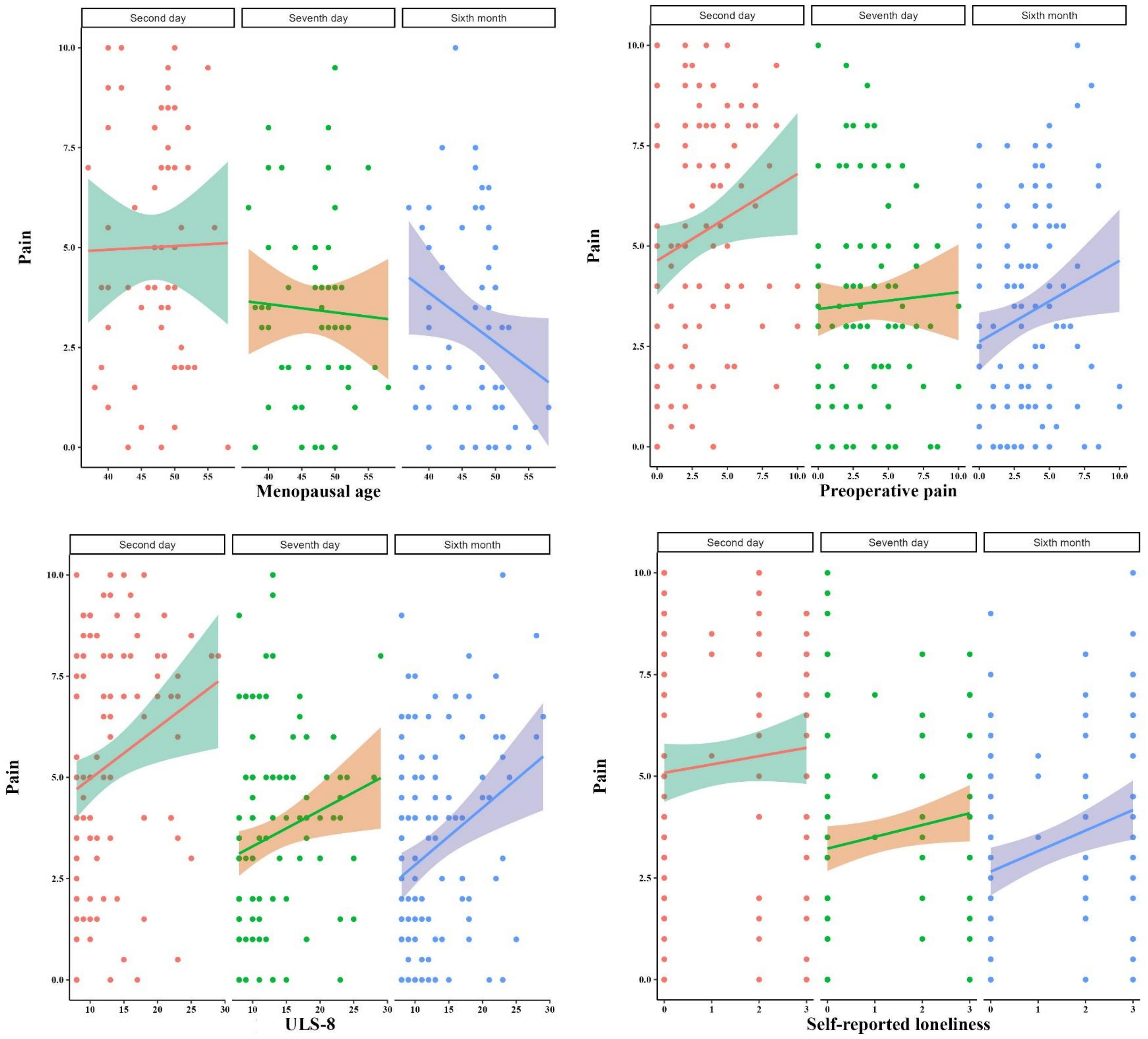

Figure S1. (Continue) The relation between pain in different times (second day, seventh day, and sixth month) and variables using the linear method.

Table S2. The longitudinal impact of variables on the pain during the time

**Variable**	**Estimate (std. error)**	**P-value**
Age	-0.01 (0.04)	0.694
Time	-0.68 (0.64)	0.287
Age : Time	-0.01 (0.01)	0.573
BMI	-0.04 (0.09)	0.634
Time	-0.98 (0.92)	0.293
BMI : Time	0.00 (0.03)	0.953
Menopausal age	0.09 (0.12)	0.451
Time	2.14 (2.02)	0.294
Menopausal age : Time	-0.07 (0.04)	0.125
Preoperative pain	0.18 (0.15)	0.236
Time	-1.00 (0.23)	<0.001
Preoperative pain : Time	-0.01 (0.06)	0.869
Expected postoperative pain	0.40 (0.18)	0.025
Time	-0.49 (0.41)	0.241
Expected postoperative pain : Time	-0.10 (0.07)	0.151
HADS	0.30 (0.15)	0.047
Time	-0.45 (0.51)	0.379
HADS : Time	-0.07 (0.06)	0.240
ULS-8	0.11 (0.07)	0.102
Time	-1.12 (0.37)	0.003
ULS8 : Time	0.01 (0.03)	0.802
Self-Reported Loneliness	0.12 (0.28)	0.664
Time	-1.19 (0.19)	<0.001
Self-Reported Loneliness : Time	0.13 (0.11)	0.205
Education Upper-diploma	-0.27 (0.76)	0.720
Time	-1.02 (0.18)	<0.001
Education Upper-diploma : Time	-0.04 (0.29)	0.898
Marital Status, Married	0.46 (1.13)	0.683
Time	-0.98 (0.40)	0.014
Marital Status, Married : Time	-0.05 (0.42)	0.903
No. of Children	0.39 (0.34)	0.252
Time	-0.50 (0.40)	0.211
No of Children : Time	-0.18 (0.13)	0.150
Previous breast operation Yes	0.50 (0.96)	0.601
Time	-0.98 (0.16)	<0.001
Previous breast operation Yes : Time	-0.28 (0.36)	0.446
Neoadjuvant chemotherapy Yes	-2.30 (0.82)	0.006
Time	-1.19 (0.17)	<0.001
Neoadjuvant chemotherapy Yes : Time	0.55 (0.31)	0.079
Low back pain Yes	2.36 (1.06)	0.027

Time	-0.97 (0.16)	<0.001
Low back pain Yes : Time	-0.43 (0.40)	0.281
Knee pain Yes	0.67 (1.03)	0.516
Time	-1.03 (0.16)	<0.001
Knee pain Yes : Time	-0.03 (0.39)	0.948
Headache Yes	0.46 (0.92)	0.618
Time	-1.09 (0.16)	<0.001
Headache Yes : Time	0.30 (0.34)	0.387
Chronic pain Yes	1.07 (0.77)	0.169
Time	-1.01 (0.19)	<0.001
Chronic pain Yes : Time	-0.05 (0.29)	0.873
Diabetes mellitus Yes	1.10 (1.08)	0.313
Time	-0.97 (0.15)	<0.001
Diabetes mellitus Yes : Time	-0.44 (0.42)	0.288
Hypertension Yes	-0.41 (1.24)	0.742
Time	-1.04 (0.15)	0.000
Hypertension Yes : Time	0.05 (0.46)	0.907
Breast surgery; Mastectomy	0.65 (0.94)	0.490
Breast surgery; Other	-0.12 (1.06)	0.909
Time	-0.89 (0.19)	<0.001
Breast surgery; Mastectomy : Time	-0.25 (0.35)	0.482
Breast surgery; Other : Time	-0.56 (0.40)	0.160
Axillary surgery; ALND	1.13 (0.58)	0.051
Time	-0.90 (0.24)	<0.001
Axillary surgery; ALND: Time	-0.48 (0.31)	0.123
Pathology; Malignant	0.29 (1.05)	0.782
Time	-1.47 (0.36)	<0.001
Pathology; Malignant : Time	0.53 (0.39)	0.183
Analgesic use Yes	3.64 (0.82)	<0.001
Time	-0.83 (0.17)	<0.001
Analgesic use Yes : Time	**-0.74 (0.33)**	**0.025**
Aromatase inhibitor Yes	1.39 (1.16)	0.233
Time	-0.99 (0.15)	<0.001
Aromatase inhibitor Yes : Time	-0.29 (0.44)	0.506
SERM Yes	0.24 (0.77)	0.760
Time	-1.08 (0.19)	<0.001
SERM Yes : Time	0.11 (0.29)	0.698
Adjuvant chemotherapy Yes	2.12 (0.73)	0.004
Time	-0.82 (0.19)	<0.001
Adjuvant chemotherapy Yes : Time	-0.46 (0.28)	0.106
Adjuvant radiotherapy; Yes	-1.80 (0.74)	0.017
Time	-1.53 (0.19)	<0.001
Adjuvant radiotherapy; Yes : Time	1.00 (0.27)	**<0.001**

Abbreviation: Body mass index (BMI), Hospital Anxiety and Depression Scale (HADS), University of California Los Angeles (UCLA) Loneliness Scale (ULS) questionnaire with eight items (ULS-8), Axillary lymph node dissection (ALND), Selective estrogen receptor modulator (SERM). The mixed effect model was used to evaluate the changes in the impact of variables on pain over time.

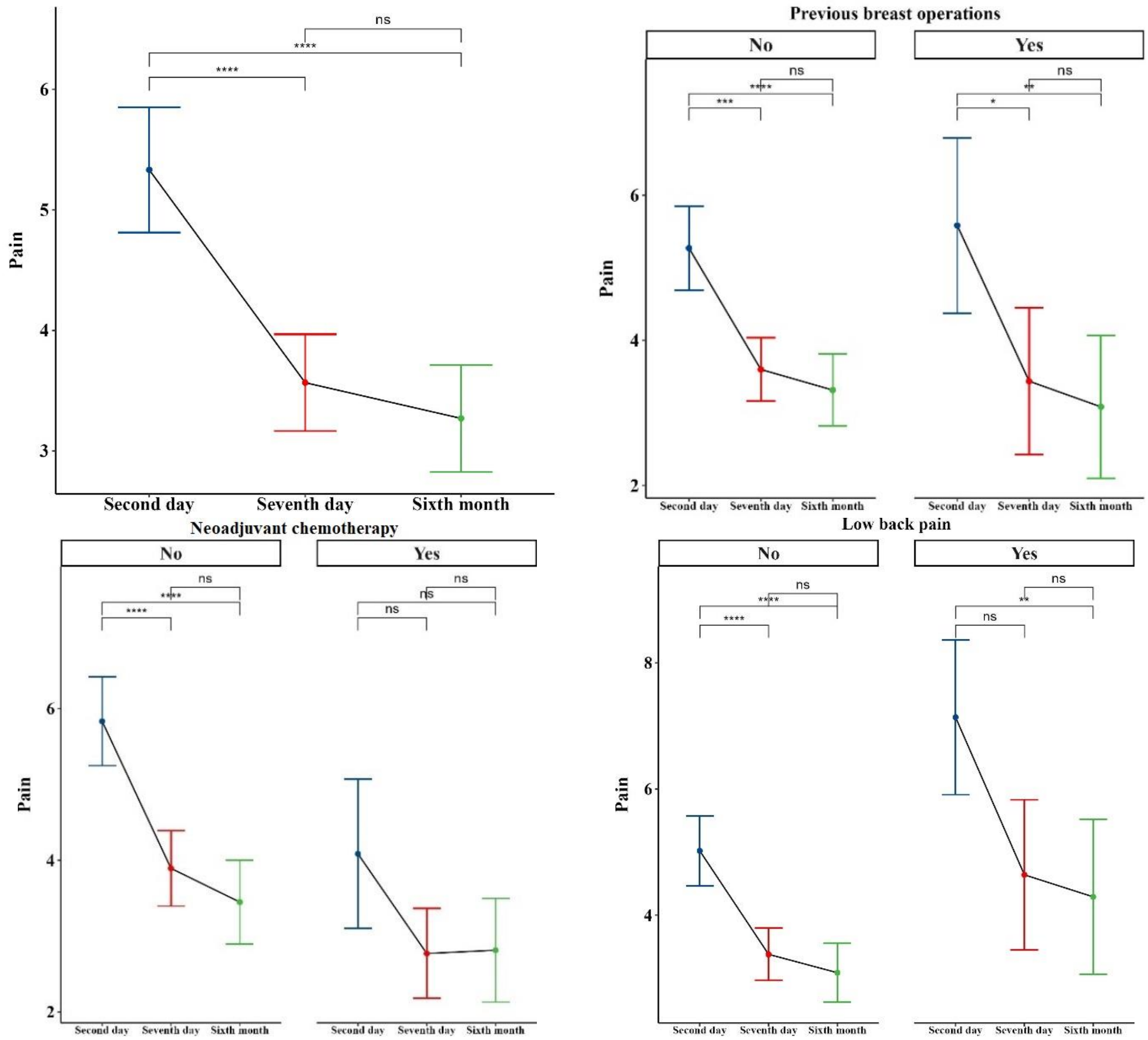

Figure S2. Line plot of pain during time points by different variables

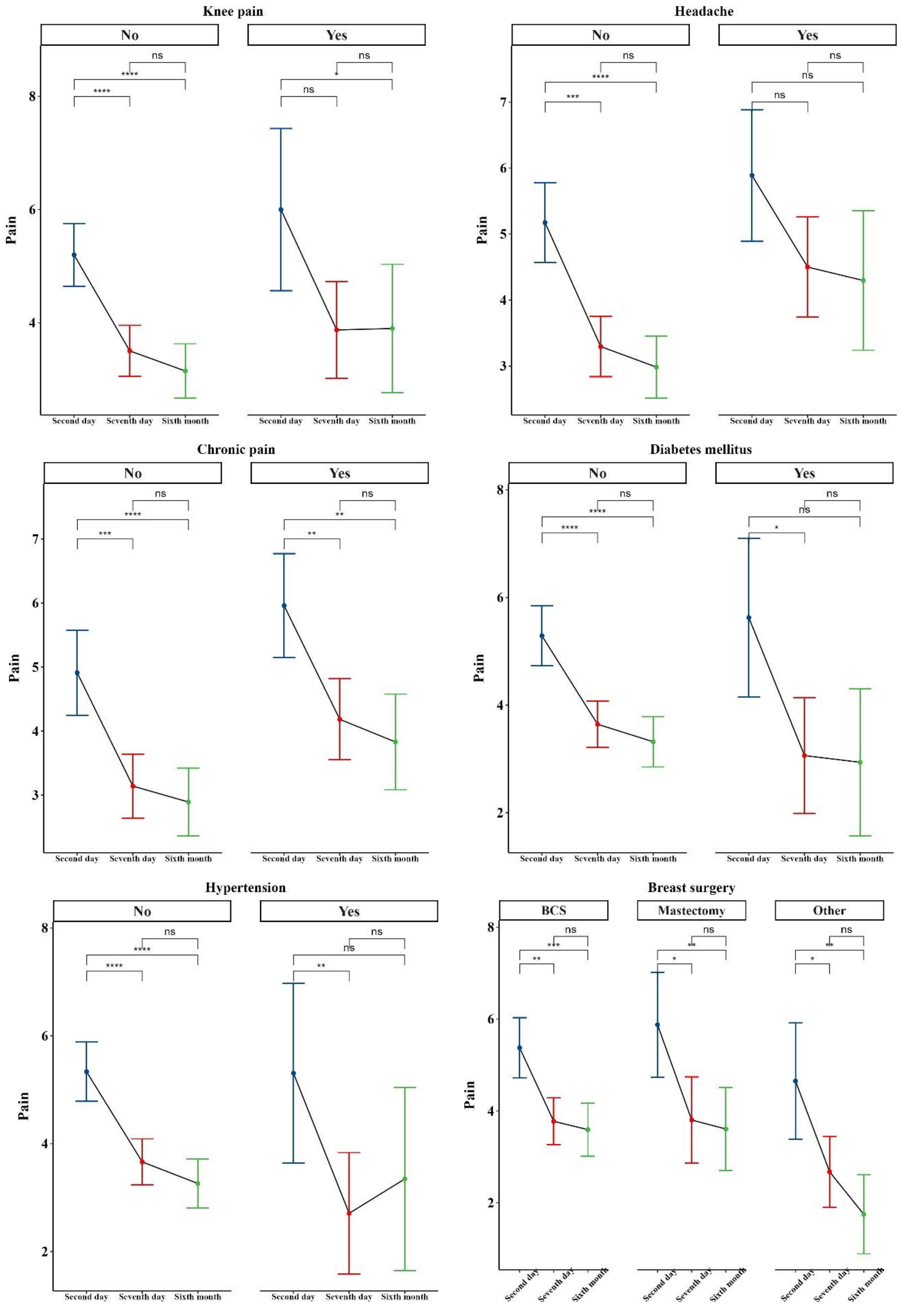

Figure S2. (Continue) line plot of pain during time points by different variables

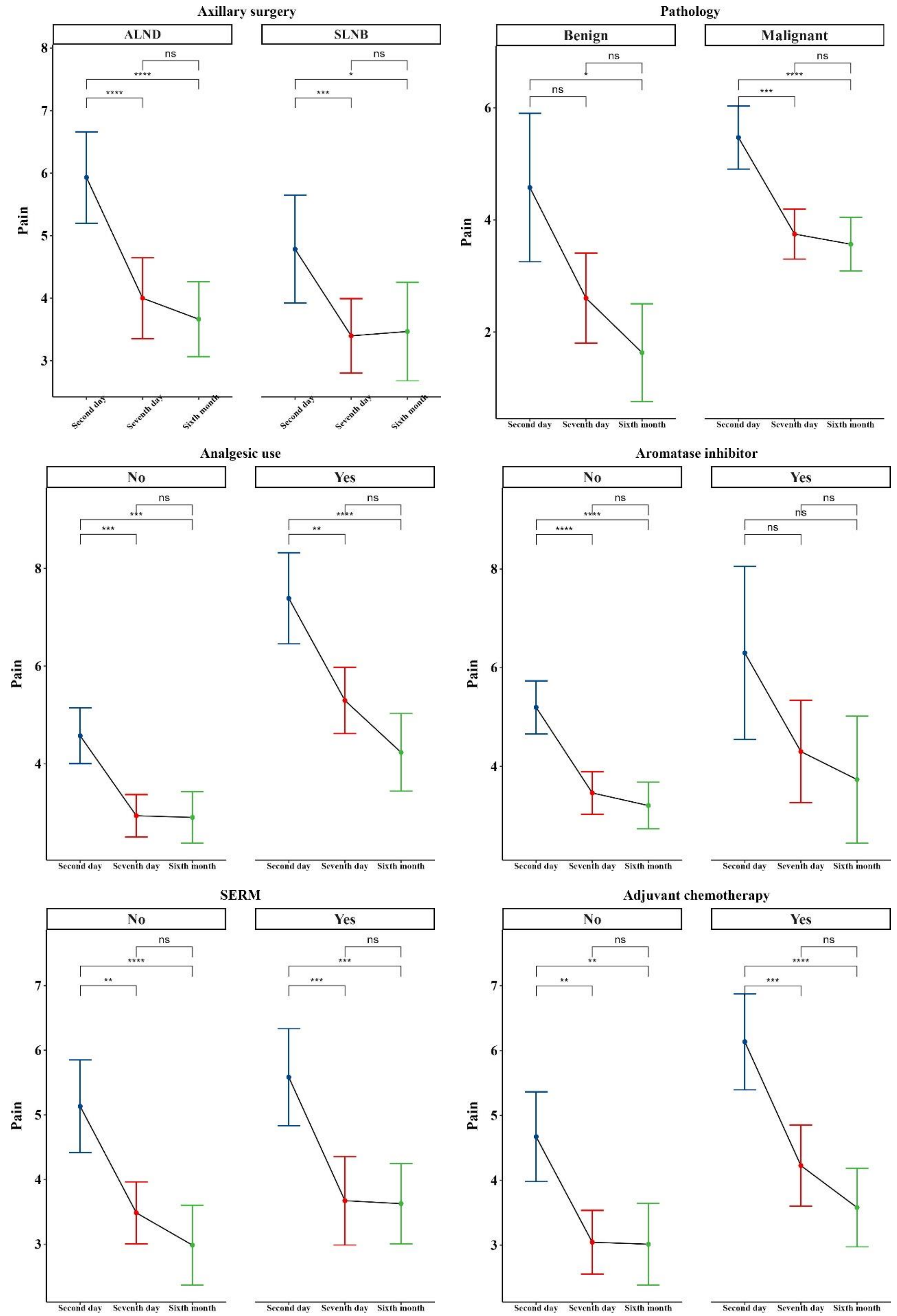

Figure S2. (Continue) line plot of pain during time points by different variables

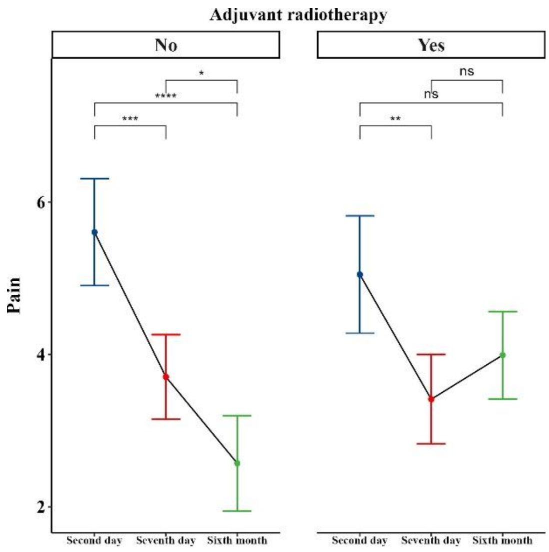

Figure S2. (Continue) line plot of pain during time points by different variables

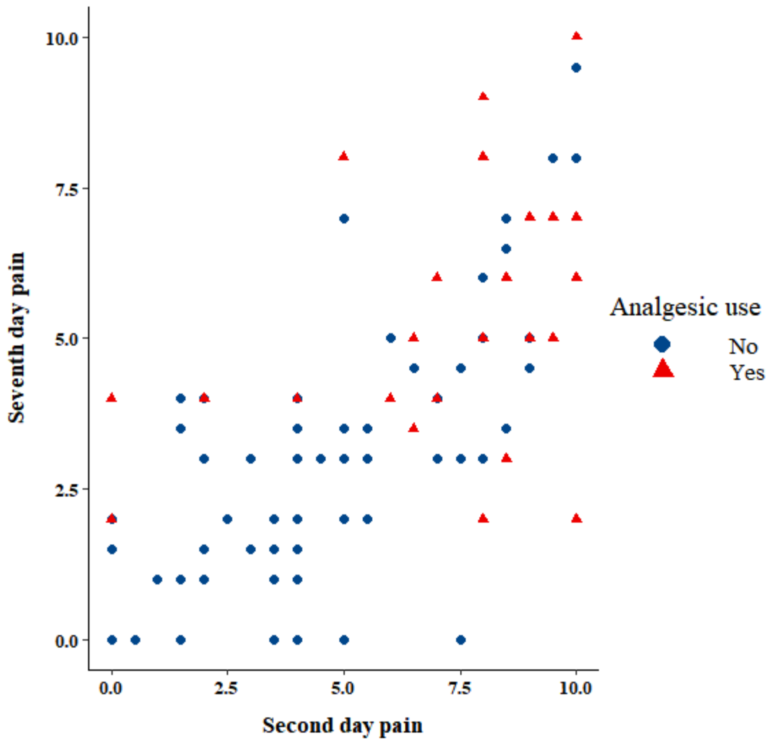

Figure S3. The scatter plot of pain on the second and seventh days by analgesic use.
